# Large Aggressive Angiomyxoma of the Liver: A Case Report and Brief Review of the Literature

**DOI:** 10.3389/fonc.2019.00133

**Published:** 2019-03-08

**Authors:** Pi-Jiang Sun, Yan-Hua Yu, Xi-Jun Cui

**Affiliations:** ^1^Department of Hepatobiliary Surgery, Weihai Central Hospital, Weihai, China; ^2^Department of Dermatology, Weihai Central Hospital, Weihai, China

**Keywords:** aggressive angiomyxoma, liver tumor, rare tumor, hepatectomy, immunohistochemistry

## Abstract

Aggressive angiomyxoma (AAM) is an uncommon mesenchymal myxoid tumor that almost solely involves the soft tissues of the perineum and pelvis. An AAM originating from the liver is extremely rare. Herein, we present a case of a 45-year-old female with a large mass in the left lateral lobe of the liver. She underwent a left lateral lobe hepatectomy. The histopathology of the resected specimen showed features that were characteristic of AAM. Immunohistochemical analysis of the neoplastic cells showed reactions to antibodies against CD34, smooth muscle actin (SMA), and Ki67 (2%) and showed no reactions to antibodies against Estrogen receptor (ER), C-keratin (CK), and Desmin. The patient was subsequently diagnosed with a primary AAM of the liver. This is the largest AAM of the liver that has been reported. We hereby report these findings and review the current literature.

## Introduction

Aggressive angiomyxoma (AAM) is a rare, infiltrative tumor composed of bland spindle cells in a myxoid background studded with blood vessels of varying calibers. It is found almost solely in the soft tissues of the female pelvis and perineum. An AAM originating from the liver is extremely rare. In fact, only three cases of AAM occurrences in the liver have been reported worldwide. In this paper, we report a large AAM of the liver. To the best of our knowledge, we report the largest AAM that has been found in the liver.

## Case Presentation

**Figure 1:**
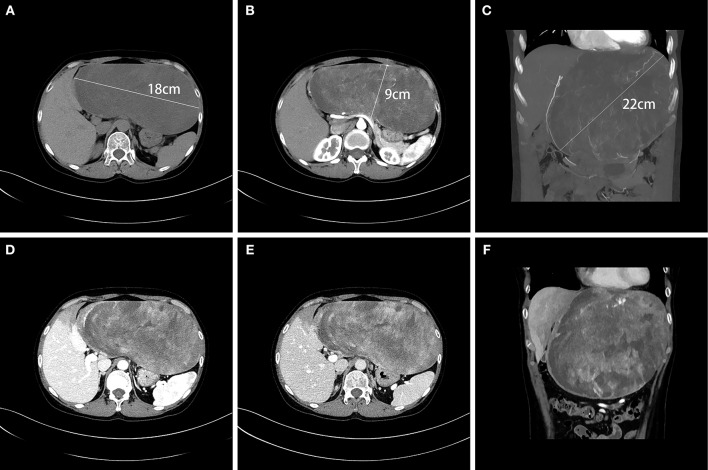
CT showed a large, regular, hypodense 22 × 18 × 9 cm mass in the left lateral lobe of the liver **(A–C)**. Blood vessels were observed in the tumor in the arterial phase **(B,C)**, and the tumor was progressively unevenly enhanced in the portal vein phase **(D)** and in the delayed phase **(E)**. The liver tissue adjacent to the tumor was weakly enhanced **(F)**.

**Figure 2:**
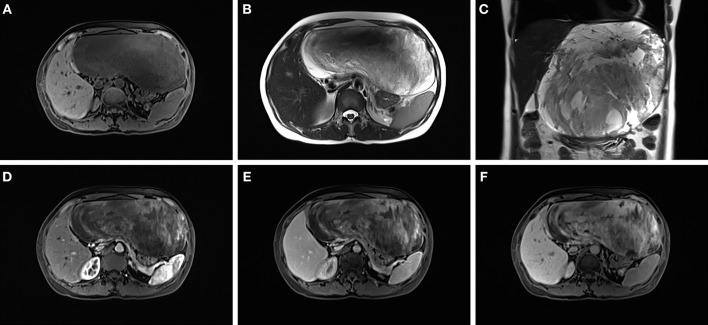
The lesion showed a slightly decreased signal intensity on T1-weighted images **(A)** and a slightly increased signal intensity on T2-weighted images **(B,C)**. A progressive uneven enhancement was observed after the infusion of contrast material **(D–F)**.

**Figure 3:**
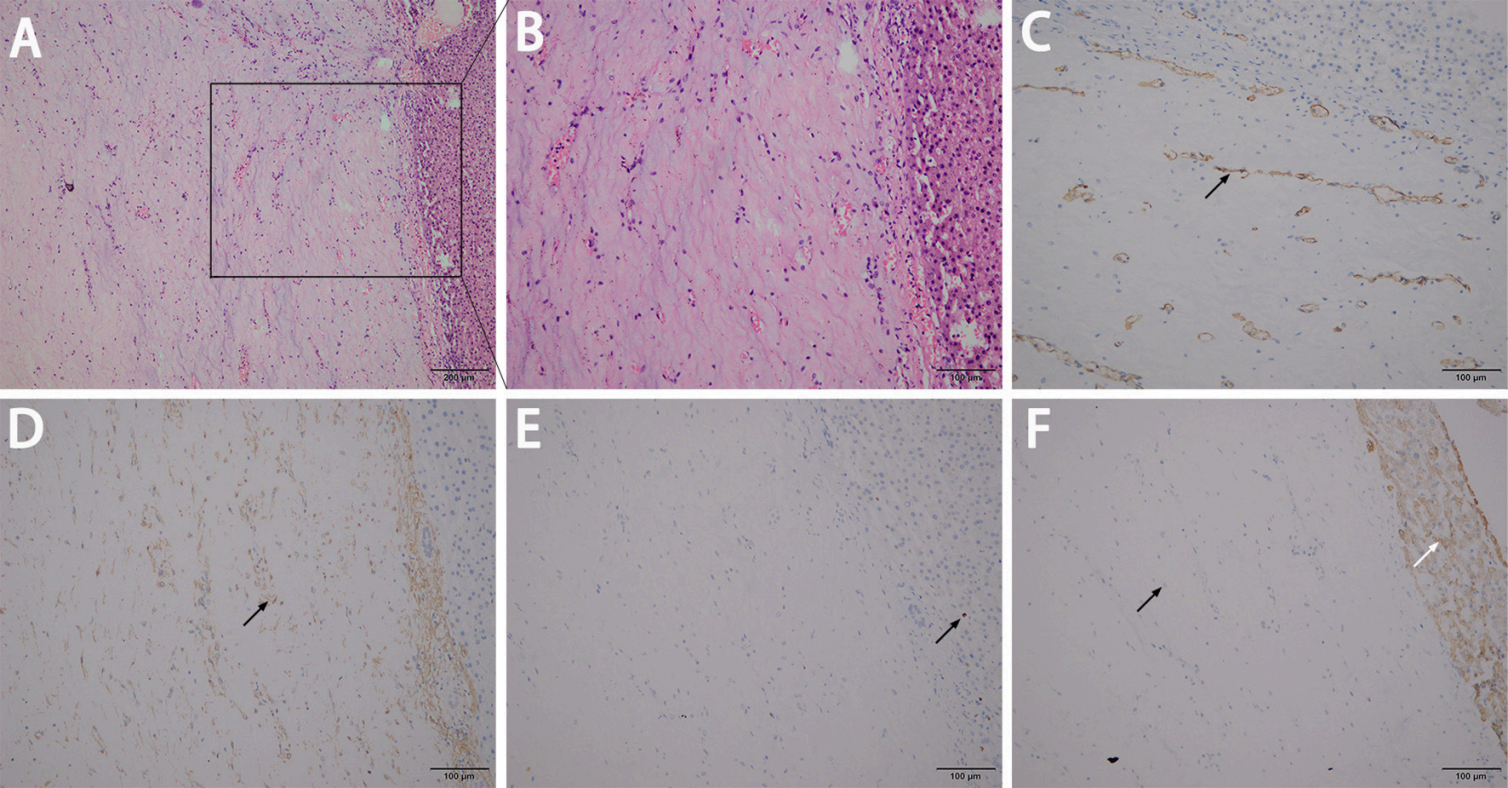
A microscopic examination revealed that the tumor consisted of spindle cells with vascular proliferation in the myxoid stroma **(A,B)**. Immunohistochemistry showed strong and diffuse staining for CD34 **(C)**, SMA **(D)**, and Ki67 at ~2% **(E)**, and showed more negative staining in the neoplastic cells (left black arrow) but positive the in normal liver cells (right white arrow) for CK **(F)**.

A 45-year-old female presented to our hospital with a 5-month history of an epigastric mass. She had no history of carcinoma. A physical examination revealed a 20 × 18 cm palpable mass in the left-middle-upper abdomen. The laboratory findings, including tumor markers, were all within the normal ranges. Abdominal computed tomography (CT) showed a large 22 × 18 × 9 cm regular hypodense mass in the left lateral lobe of the liver that was progressively enhanced after infusion of contrast material, with blood vessels observed in the tumor (Figure 1). Magnetic resonance imaging (MRI) revealed a regular heterogeneous mass of 22 × 18 × 9 cm in the left lateral lobe of the liver. The lesion showed a slightly decreased signal intensity on T1-weighted images and a slightly increased signal intensity on T2-weighted images. Progressive, uneven enhancement was observed (Figure 2). Preoperatively, we regarded the lesion as either an angiosarcoma or hemangioma. Subsequently, a left lateral lobe hepatectomy was performed with no complications. The postoperative recovery was uneventful, and the patient was discharged 7 days after surgery. A macroscopic examination showed a 22 × 18 × 9 cm tumor that was oval, well circumscribed, and soft. The cut surface was whitish, grayish red, with some areas of the tumor being cystic and containing myxoid components. A microscopic examination revealed that the tumor consisted of spindle cells, with vascular proliferation in the myxoid stroma. Immunohistochemistry showed strong and diffuse staining for CD34, smooth muscle actin (SMA) and Ki67 (2%) and negative staining for Estrogen receptor (ER), C-keratin (CK), and Desmin (Figure 3). The patient was diagnosed with a hepatic AAM instead of a sarcoma or a cavernous hemangioma. The patient was postoperatively monitored for 18 months, and there were no signs of recurrence or metastasis.

## Discussion

AAM predominantly occurs in adult females of reproductive age and was first described as a separate histopathological entity in 1983 (1). We summarized the available cases that were reported previously, with only 3 cases of AAM occurrence in the liver having been reported (Table 1) (2–4). All of the patients were female, and the age range of these patients was 33–50 years old. Because of the low incidence, it is difficult to preoperatively confirm the diagnosis. All cases were confirmed based on postoperative histopathologic specimens. Immunohistochemical studies showed positive detections for vimentin, Desmin, ER and Muscle-specific actin (MSA), with SMA, CD34, factor XIIIa possibly showing positive detections in some cases, whereas S-100 and CK are not expressed by neoplastic cells (5). Cytogenetic analysis and fluorescent *in situ* hybridization have confirmed the HMGA2 gene as the target of the 12q rearrangements in AAM (6, 7). But McCluggage et al. found that HMGA2 was negative in only 2 out of 12 cases of AAM, prompted that HMGA2 is a sensitive but not specific immunohistochemical marker of AAM (8). The ideal treatment for patients with AAM is a complete, wide surgical resection because AAM is regarded as a locally aggressive neoplasm; its local recurrence rate can be as high as 70%, with most recurrences occurring within 2 years, but some recurrences may also occur as early as a few months or as late as 20 years post-resection (9, 10). Distant metastases have also been reported, but they are extremely rare (11–13). All of the hepatic AAM cases that have been reported have found no recurrences during the postoperative follow-ups, possibly because it is easier to completely remove the tumor when the AAM is located in the liver.

**Table 1:** The previously reported cases of aggressive angiomyxoma of the liver.

**References**	**Age/sex**	**Size**	**Postoperatively follow**	**Immunohistochemical profile**
Qi et al. (2)	50 F	2.0 × 2.0 × 1.0 cm	6 months	**Positive:** vimentin, CD34, SMA **Negative:** desmin, S-100, Ki-67, EMA, ER, PR, CD99, CD10, CAM5.2, and CK19
Sato et al. (3)	33 F	8.0 × 7.5cm	10 months	**Positive:** vimentin, desmin, CD34,ER, PgR **Negative:** S-100, EMA, CK19, CD99, HMB45, and α-smooth muscle actin
Malik et al. (4)	46 F	13 × 6 cm	60 months	**Positive:** smooth muscle actin **Negative:** CD31, CD34, CD117, DOG1, desmin, S100, AE1/AE3, a-fetoprotein, UC4, caldesmon, estrogen, receptor and progesterone receptor
Present case	45 F	22 × 15 × 9 cm	18 months	**Positive:** CD34, SMA, Ki-67(2%) **Negative:** desmin, S100, CK, ER

## Conclusion

In summary, we report a case of a large hepatic AAM and summarize the reported cases. Specifically, we describe the fact that an AAM originating from the liver is a rare mesenchymal myxoid tumor that has a good prognosis after surgery to remove the tumor.

## Author Contributions

X-JC: conceptualization. Y-HY: data curation. P-JS: investigation, validation, and writing of the original draft.

### Conflict of Interest Statement

The authors declare that the research was conducted in the absence of any commercial or financial relationships that could be construed as a potential conflict of interest.
